# The LncRNA Connectivity Map: Using LncRNA Signatures to Connect Small Molecules, LncRNAs, and Diseases

**DOI:** 10.1038/s41598-017-06897-3

**Published:** 2017-07-27

**Authors:** Haixiu Yang, Desi Shang, Yanjun Xu, Chunlong Zhang, Li Feng, Zeguo Sun, Xinrui Shi, Yunpeng Zhang, Junwei Han, Fei Su, Chunquan Li, Xia Li

**Affiliations:** 1 College of Bioinformatics Science and Technology, Harbin Medical University, Harbin, 150081 China; 2 Department of Medical Informatics, Daqing Campus, Harbin Medical University, Daqing, 163319 China

## Abstract

Well characterized the connections among diseases, long non-coding RNAs (lncRNAs) and drugs are important for elucidating the key roles of lncRNAs in biological mechanisms in various biological states. In this study, we constructed a database called LNCmap (LncRNA Connectivity Map), available at http://www.bio-bigdata.com/LNCmap/, to establish the correlations among diseases, physiological processes, and the action of small molecule therapeutics by attempting to describe all biological states in terms of lncRNA signatures. By reannotating the microarray data from the Connectivity Map database, the LNCmap obtained 237 lncRNA signatures of 5916 instances corresponding to 1262 small molecular drugs. We provided a user-friendly interface for the convenient browsing, retrieval and download of the database, including detailed information and the associations of drugs and corresponding affected lncRNAs. Additionally, we developed two enrichment analysis methods for users to identify candidate drugs for a particular disease by inputting the corresponding lncRNA expression profiles or an associated lncRNA list and then comparing them to the lncRNA signatures in our database. Overall, LNCmap could significantly improve our understanding of the biological roles of lncRNAs and provide a unique resource to reveal the connections among drugs, lncRNAs and diseases.

## Introduction

Long non-coding RNAs (lncRNAs) are transcripts that are longer than 200 nucleotides and are not translated into proteins. Recently, a large number of lncRNAs have been identified, and increasing evidence shows that lncRNAs play critical roles in various biological processes and are engaged in multiple biological mechanisms^1–3^, such as physiological, chromatin modification, transcriptional/post-transcriptional regulation and human diseases^4^. Aberrant expressions of lncRNAs were thought to play critical roles in the progression and development of various cancer types, some of which could be further evaluated as potential biomarkers. Further, the expressions of lncRNAs would change when treated with bioactive small molecules. For example, the expression of lncRNA GAS5 was decreased in SKBR-3/Tr cells and breast cancer tissue from trastuzumab-treated patients^5^, and Lavorgna *et al*. proposed that lncRNAs may be a new class of therapeutic target, especially in cancers^6^. Therefore, lncRNAs could be considered genomic signatures for discovering the “connections” between drugs and diseases.

Constructing a database to characterize and establish the connections among diseases, lncRNAs and drugs is a meaningful endeavor. Previously, RNA-seq was the only comprehensive way to profile lncRNA expression. However, because of the high cost associated with the use of this technique, publically available RNA-seq data sets induced by small molecules are relatively limited compared to array-based expression profiles. In contrast, the Connectivity Map has a large number of array-based gene-expression profiles from cultured human cells that have been treated with bioactive small molecules. Although lncRNAs are not the intended targets of measurement in the original array design, microarray probes can be reannotated for interrogating the lncRNA expression^1, 7, 8^. By repurposing microarray data from the Connectivity Map database for probing lncRNA expression, we constructed a database called LNCmap to characterize lncRNA signatures of drugs, and establish the correlations among diseases, lncRNAs, and the action of small molecule therapeutics. In the LNCmap database, we repurposed a total of 5916 Affymetrix microarray raw data instances and obtained 237 lncRNAs signatures of up to 1262 small molecular drugs. The LNCmap provided a user-friendly interface for the convenient browsing, retrieval and download the dataset. Additionally, we also provided two pattern-matching tools to establish the connections between diseases and drugs in terms of lncRNAs.

## Materials and Methods

### Data sources

We downloaded raw data files from the Connectivity Map database (http://www.broadinstitute.org/cmap/)^9^, and the data referred to three different platforms (HG-U133A, HT_HG-U133A, HT_HG-U133A_EA). We obtained 5916 Affymetrix microarrays (.CEL files) corresponding to 1262 bioactive small molecules profiled by two different Affymetrix microarray platforms: Human Genome U133 Set (HG-U133A) and GeneChip HT Human Genome U133 Array Plate Set (HT_HG-U133A), which contained 674 and 5242 instances respectively. Due to the absent sequence files of HT_HG-U133A_EA platform, 184 instances from this chipset were not used in our work. Drug information (such as ATC code) was obtained from the DRUGBANK database (http://www.drugbank.ca/) and KEGG drug (http://www.kegg.jp/kegg/drug/).

### Repurposing microarray data for probing lncRNA vexpression

We developed a similar computational method to repurpose microarray data for probing lncRNA expression according to the pipeline of ncFANs^7, 10^. The ncFANs proposed by Liao *et al*. has been widely used for the functional annotation of long non-coding RNAs in various studies^10–13^, and becomes a popular method to re-annotate microarray data to obtain high throughput lncRNA expression profiles. We first collected lncRNA transcript sequences from GenCode (gencodeV19), and we used BLASTn to align the probe sequences provided by Affymetrix (http://www.affymetrix.com) to lncRNA transcript sequences. Alignment results with e-value greater than 10^−6^ were removed, and we filtered the alignment results as follows: (i) set alignment_length to 25, and probes that perfectly matched to a transcript with no mismatch were retained; (ii) all probes that targeted both lncRNA and protein-coding transcripts were removed; (iii) all lncRNA transcripts corresponding to retained probes were mapped to the genome and annotated at the gene level; and (iv) lncRNA genes matched by fewer than three probes were discarded. After these filtering steps, we used the R package affy to compute expression values for all of the Cmap instance samples and obtained log2fold change values between the treatment samples and the corresponding control samples. Finally, from these two platforms, we obtained expression values for 237 lncRNAs that were affected by 1262 drugs.

### Enrichment analysis

Based on the correlations between drugs and lncRNAs in the LNCmap database, users can identify candidate drugs for a particular disease by inputting the corresponding lncRNA expression profiles or an associated lncRNA list and then comparing them to drug-induced lncRNA sets (mentioned in Database content). To do this, we provided two analysis strategies, LncRNA Set Enrichment Analysis (LSEA) and Over-Representation Analysis (ORA), to establish connections between diseases and drugs in terms of lncRNAs.

### LSEA

Although lncRNAs were thought to elucidate the underlying biological mechanisms in various biological states, such as disease, or induced with a variety of chemicals. However, the connections among diseases, lncRNAs and drugs are not well characterized. Here, we introduce a novel method, called lncRNA-set enrichment analysis (LSEA), to identify the drugs’ mode-of-action (MoA) based on lncRNA expression and establish the correlations among lncRNAs, drugs and diseases.

The inputs of LSEA were the lncRNA expression profile and the label file of a disease, in which samples should be classified into two classes (such as normal and disease), labeled 0 or 1, respectively. Following the pipeline of the Gene Set Enrichment Analysis method^14^, in LSEA, we obtained a ranked list L of lncRNAs by computing the lncRNA expression values, and we calculated an enrichment score (ESi) for each drug-induced lncRNA set i as follows: by walking down the list L, we increased the running-sum statistic when we encountered a lncRNA that was in drug-induced lncRNA set i, and decreased it when we encountered lncRNAs that were not in set i, ESi was the maximum deviation from zero encountered in the random walk. Given a query lncRNA expression profiles, LSEA checked for each drug-induced lncRNA set whether lncRNAs of this set tended to be significantly ranked at the top (or bottom) of the list. This method derived its power by focusing on lncRNA sets, which were likely to be affected by the same drug. LSEA can be considered another type of GSEA: in GSEA, each pathway is considered a set of genes; in LSEA, the lncRNA is considered a “gene” and each drug-induced lncRNA set is considered as a “pathway”. The output of LSEA was a ranked list of drug-induced lncRNA sets represented by drug names.

### ORA

We developed another method to establish the connections between diseases and drugs based on the list of lncRNAs, according to the classic over-representation analysis (ORA). This could assess the statistical overrepresentation between a user-defined, pre-selected lncRNA list of interest and reference drug-induced lncRNA sets. The input of ORA was a list of lncRNAs (e.g., differentially expressed lncRNAs related to a special disease), and the hypergeometric test was used to calculate the statistical significance for each drug-induced lncRNA set. The p-value can be calculated to evaluate the enrichment significance for each lncRNA set as follows:

$${\rm{p}}=1-\sum _{{\rm{x}}=0}^{{\rm{r}}-1}\frac{(\begin{array}{c}{\rm{t}}\\ {\rm{x}}\end{array})(\begin{array}{c}{\rm{m}}-{\rm{t}}\\ {\rm{n}}-{\rm{x}}\end{array})}{(\begin{array}{c}{\rm{m}}\\ {\rm{n}}\end{array})}$$

Here, we collected *m* total lncRNAs, of which *t* were involved in the drug-induced lncRNA set, and the input lncRNA list contained *n* lncRNAs, of which *r* were involved in the drug-induced lncRNA set. After calculating the p-value, we adopted the FDR-corrected q-values to reduce the false positive discovery rate. The output of ORA was a ranked list of drug-induced lncRNA sets represented by drug names.

## Results

### Database content

**Figure 1:**
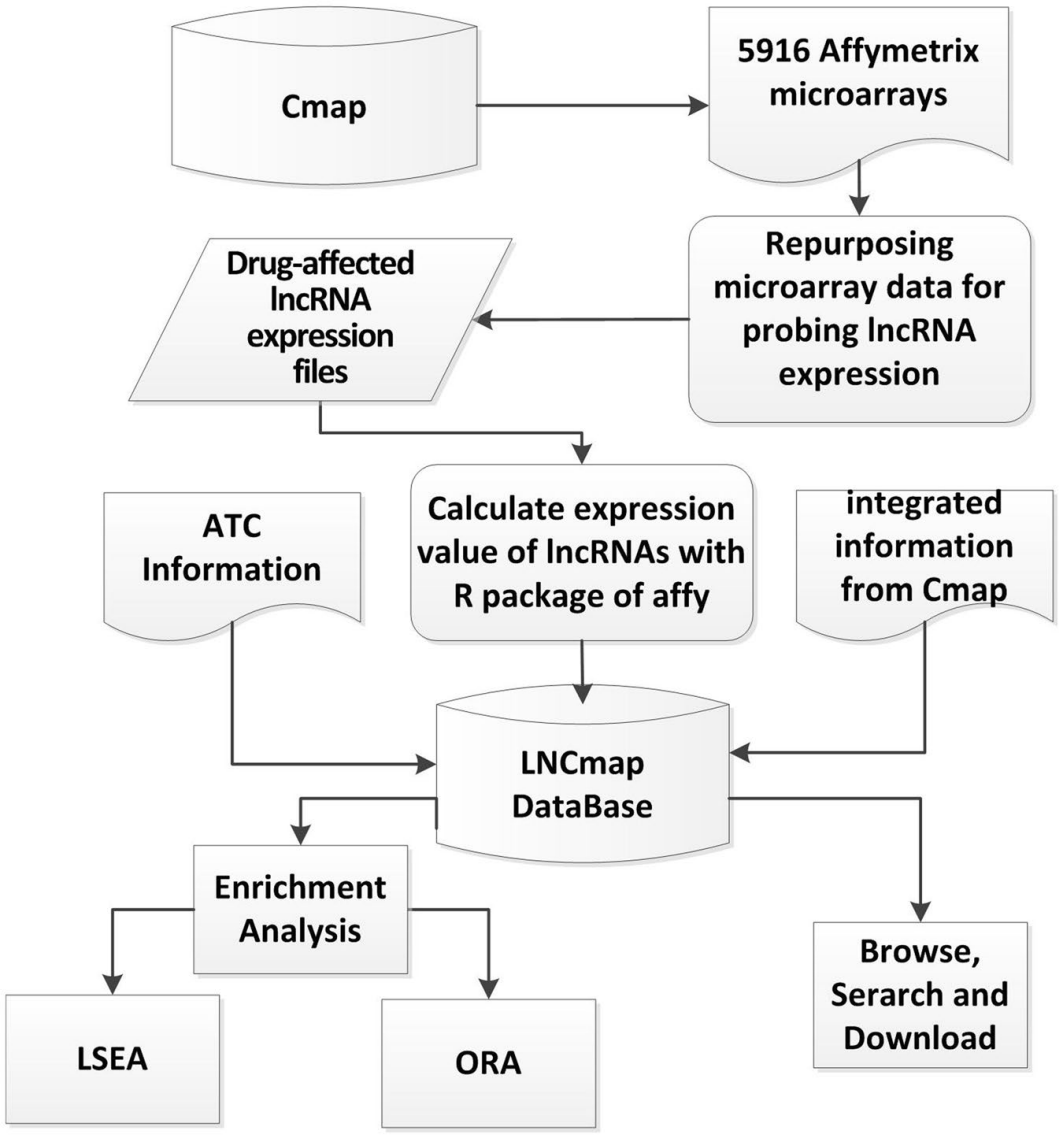
Schematic data flowchart of LNCmap.

The LNCmap was designed to establish the connections among diseases, lncRNAs and drugs. The flowchart of the LNCmap is shown in Fig. 1. We first downloaded the raw data from the Connectivity Map database. By reannotating the microarray data for lncRNAs, we obtained the lncRNA expression profiles that had been treated with small molecular drugs. Then, we matched the perturbation and control pairs of expression profiles for each instance (experiment) according to the instances description file “cmap_instances_02.xls” and calculated log2fold change values between the treatment samples and the corresponding control samples for each instance. We provided a flexible threshold to define differentially expressed lncRNAs (DELs), which can be considered drug-affected lncRNAs. With fold change ≥2 (or fold change ≤1/2), we obtained 173 lncRNAs that were affected by 1005 small molecular drugs, corresponding to 2147 instances, and with fold change ≥1.5 (or fold change ≤2/3), we obtained 237 lncRNAs and 5523 instances belonging to 1262 small molecular drugs. All of the drugs and affected lncRNAs were restored in the LNCmap database according to the original instance ID. Additionally, we collected the classification information from the Anatomical Therapeutic Chemical (ATC) classification for these small molecular drugs, and we provided integrated information, such as the drug name, lncRNA Ensemble ID, log2fold change values and instance ID. The LNCmap provided a user-friendly interface to implement retrieve, browse and download functions based on these data. Additionally, the drug-affected lncRNAs were merged if the corresponding instances belonged to the same drug (bioactive small molecule); these lncRNAs were defined as *drug-induced lncRNA sets*, which were also restored in the LNCmap database and used for LSEA and ORA enrichment analysis.

### Enrichment analysis

We developed two enrichment analysis algorithms (LSEA and ORA) to establish the connections between diseases and drugs in terms of lncRNAs.

**Figure 2:**
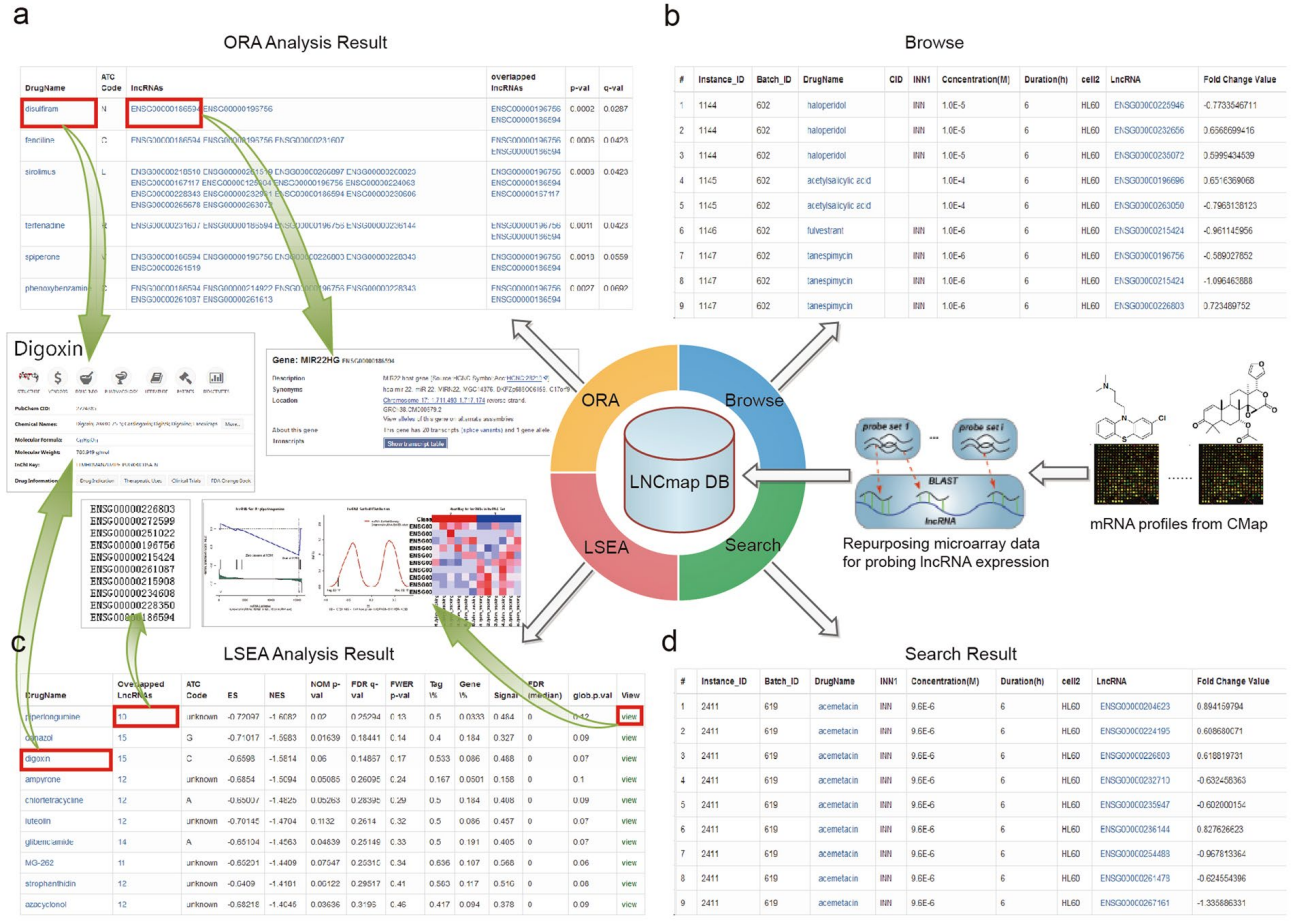
Display of LNCmap website functions. (**a**) The ORA analysis results. (**b**) The browsing of the LNCmap dataset.  (**c**) The LSEA analysis results. (**d**) The search results of LNCmap.

Users could flexibly select the LSEA or ORA method, both the results were provided as ranked list of drugs with drug-induced lncRNA sets and could be downloaded from the result page. Top-ranked drugs may be used to guide the use of drugs for disease. We used primary colorectal cancer data (SRP029880) as example to perform LSEA and ORA enrichment analysis. With the ORA method, we input a list of differentially expressed lncRNAs related to primary colorectal cancer and obtained a table (Fig. 2a; Supplementary dataset 1) that included the drug name (instance ID), ATC code (drug name), drug-induced lncRNAs, overlapped lncRNAs, p-value and FDR q-value. Drug information can be found at https://www.ncbi.nlm.nih.gov/pccompound by clicking on “DrugName” and lncRNA details can be found at http://asia.ensembl.org/ by clicking on the lncRNA hyperlink. Of top-ranked drugs, there were some known anti-cancer activity compounds. For example, disulfiram^15^ and sirolimus^16^ are used for colorectal cancer treatment, and fendiline^17^ is an anti-cancer drug that is used to treat pancreatic cancer. In the LSEA method, we input an expression profile and a label file of primary colorectal cancer, and the LSEA result was displayed as a table (Fig. 2c; Supplementary dataset 2) that contained the drug name (or instance ID) ranked by p-value, overlapped lncRNAs, ATC code (or drug name), ES, NES, normal p-value, FDR q-value, and FWER p-value. Drug information can be found at https://www.ncbi.nlm.nih.gov/pccompound by clicking on “DrugName”, overlapped lncRNAs can be found by clicking on the “number of overlapped lncRNAs” and an overview picture of compared results can be displayed by clicking on the hyperlink “view”. Detailed analysis results, including statistics, plots and report files of the significantly enriched drugs, were also provided as a zip file for users to download by clicking on the “Download Results” hyperlink in result page (see “Run Example” at http://www.bio-bigdata.com/LNCmap/lsea). Of the top-ranked drugs, apigenin^18^ is used for colorectal treatment, and lycorine^19^ is an anti-cancer drug used for prostate cancer treatment. Specially, we discovered some meaningful drug-lncRNA-disease correlations (e.g., puromycin-NEAT1-colorectal cancer). Puromycin was one of the top-ranked drugs in the LSEA results, and we verified that puromycin^20^ was used for colorectal treatment by searching the literature. We found that the expression of lncRNA NEAT1 (ENSG00000245532) in the LNCmap database significantly changed after treatment of puromycin. Meanwhile, NEAT1 was related to tumor differentiation, invasion and metastasis in colorectal cancer^21^. Furthermore, this puromycin-NEAT1-colorectal cancer correlation was verified by real-time PCR experiment (see Supplementary Fig. S1, Supplementary Information). Therefore, these results not only provided insight into drug repositioning but also helped explain the lncRNA signatures to discover the “connections” between drugs and diseases.

Additionally, we implemented KEGG (http://www.genome.jp/kegg/) pathway enrichment analysis for primary colorectal cancer example of LSEA and ORA. Pathway enrichment analysis was based on the co-expressed protein-coding genes of drug-affected lncRNAs using SubpathwayMiner tools (details of the enrichment procedure are provided in Supplementary Information). In the enrichment results (Supplementary dataset 3), many significantly enriched pathways were dysregulated in colorectal cancer cells, which further confirmed the LSEA and ORA results. For example, the MAPK signaling pathway (path:04010) regulated intrinsic resistance to the bromodomain and extra-terminal domain family proteins inhibitors in colorectal cancer^22^. Ye *et al*. found that down regulated lncRNA CLMAT3 promotes the proliferation of colorectal cancer cells by targeting regulators of the cell cycle pathway (path:04110)^23^, and low proteasome (path:03050) activity was related to treatment resistance in colorectal cancer^24^.

### Database architecture and web interface

LNCmap was implemented using the JavaEE framework and deployed on a Tomcat 6.0 web server. All database content was stored in a MySQL5 relationship database management system; the server-side was implemented with Java 1.7 scripts, and the web server was written in JSP. The LSEA algorithm was implemented using the core code of GSEA in R language. Due to its considerable running time, we chose a synchronous technology by packaging the analysis work as a backstage job and responding immediately with the job id. Users can use the linked unified resource locator (url) that contains the id to monitor the job’s completion, and the url will navigate to the result page when the job is complete. LNCmap allows users to access all of the key features of the web application through their mobile device. Here, we provided an intuitive and user-friendly interface to browse and search the database. The LNCmap browser was developed to view the drug-affected lncRNAs (Fig. 2b), their expression in log2fold change values and other instance information simultaneously, and the details of lncRNAs were provided by clicking on the lncRNA hyperlink. The LNCmap search toolkit offers various methods for querying the database. Users can acquire the drug-affected lncRNAs record by querying any given lncRNA or drug or both a lncRNA and a drug against the database. The search result is displayed by default as an overview table that summarizes the drug-affected lncRNAs and the corresponding instance information (Fig. 2d). Details of lncRNA and drug information are supported by the links in the table. Expression values are offered in fold change values as log-ratios with threshold of ±0.58 (i.e., fold change ≥1.5 or fold change ≤2/3). The complete query result data can be downloaded to local computers from the download links in the lower panel. In addition, LNCmap provided the ability to download all of the data, such as lncRNAs (Supplementary dataset 4), drugs, relationships between drug and affected lncRNAs, drug-induced lncRNA sets (Supplementary dataset 5) used for enrichment analysis, from the Download Page.

## Discussion

In this study, we constructed a database called LNCmap that established the correlations among diseases, small molecules, and lncRNA signatures. We first applied a computational method to repurpose microarray data collected from Cmap for probing lncRNA expression and identified drug-affected lncRNAs with differentially expressed values of fold change ≥2 (≤1/2) or fold change ≥1.5 (≤2/3) according to instance. Then, we merged drug-affected lncRNAs if the corresponding instances belonged to the same drug and defined as drug-induced lncRNA sets. These drug-induced lncRNA sets were then used for enrichment analysis to identify the drugs that may affect the corresponding disease. We also integrated information of instances and the ATC classification of drugs in the database and provided a user-friendly interface to freely retrieve, browse and download this information.

Our study characterized the connections of diseases, lncRNAs and drugs for the first time. To do this, we also developed two enrichment analysis algorithms (ORA and LSEA). ORA is a classic gene set enrichment analysis method. Here, we used the ORA to assess the statistical overrepresentation of a user-defined, pre-selected lncRNA list of interest in a reference list of known drug-induced lncRNA sets using the hypergeometric test. In contrast to ORA, LSEA incorporates expression level measurements and provides different analysis results. The enrichment analysis results showed candidate drugs for particular disease. If users were interested with some drugs and lncRNAs, they can further verify the result by experiments (e.g., quantitative real-time PCR). Users can flexibly select any methods to analyze the lncRNAs of interest with different demands.

We also noticed that there were some limitations of our current study. Compared to the tens of thousands of lncRNAs that have been found, we obtained only 237 drug-affected lncRNAs, and the number of lncRNAs in our database is thus limited. This is because the lncRNA expression was probed from traditional HG-U133A and HT_HG-U133A Affymetrix microarray platforms, from which only hundreds of lncRNAs could be reannotated. Although next-generation sequencing could identify many more lncRNAs, the publically available RNA-seq data sets induced by small molecules are relatively limited. With the development of pharmacogenomics, sequencing drug-induced lncRNA data are increasing, which will lead to increase in the quantity of drug-induced lncRNAs and more accurate correlations among small molecules and lncRNAs. Therefore, our study may be greatly improved with the development of pharmacogenomics sequencing.

## Electronic supplementary material

Supplementary Information

SI dataset 1

SI dataset 2

SI dataset 3

SI dataset 4

SI dataset 5
