# Supplementary material for: The LncRNA Connectivity Map: Using LncRNA Signatures to Connect Small Molecules, LncRNAs, and Diseases
Source: Sci Rep. 2017 Jul 27;7:6655. doi: 10.1038/s41598-017-06897-3 (PMC5532316; doi:10.1038/s41598-017-06897-3)
Supplement: Supplementary file 1 — Supplementary Information [file 41598_2017_6897_MOESM1_ESM.pdf]

The LncRNA Connectivity Map: Using LncRNA Signatures to Connect Small Molecules, LncRNAs, and Diseases

Haixiu Yang[1,†], Desi Shang[1,†], Yanjun Xu[1], Chunlong Zhang[1], Li Feng[1], Zeguo Sun[1], Xinrui Shi[1], Yunpeng Zhang[1], Junwei Han[1], Fei Su[1], Chunquan Li[2,*] and Xia Li[1,*]

[1]College of Bioinformatics Science and Technology, Harbin Medical University, Harbin, 150081, China.

[2]Department of Medical Informatics, Daqing Campus, Harbin Medical University, Daqing, 163319, China.

[†]These authors contributed equally to this work

[*]Corresponding author

Xia Li, lixia@hrbmu.edu.cn.

College of Bioinformatics Science and Technology, Harbin Medical University, Harbin, 150081, China.

Chunquan Li, lcqbio@aliyun.com

Department of Medical Informatics, Daqing Campus, Harbin Medical University, Daqing, 163319, China.

Validation of correlations among puromycin-NEAT1-colorectal cancer by PCR experiment

We validated correlations among puromycin-NEAT1-colorectal cancer, one case of enrichment results, by implementing a real-time PCR experiment and flow cytometry. The experiment results showed that the expression of lncRNA NEAT1 changed after treatment of puromycin, and induced apoptosis of cells, which indicated the enrichment results (see Supplementary Figure S1).

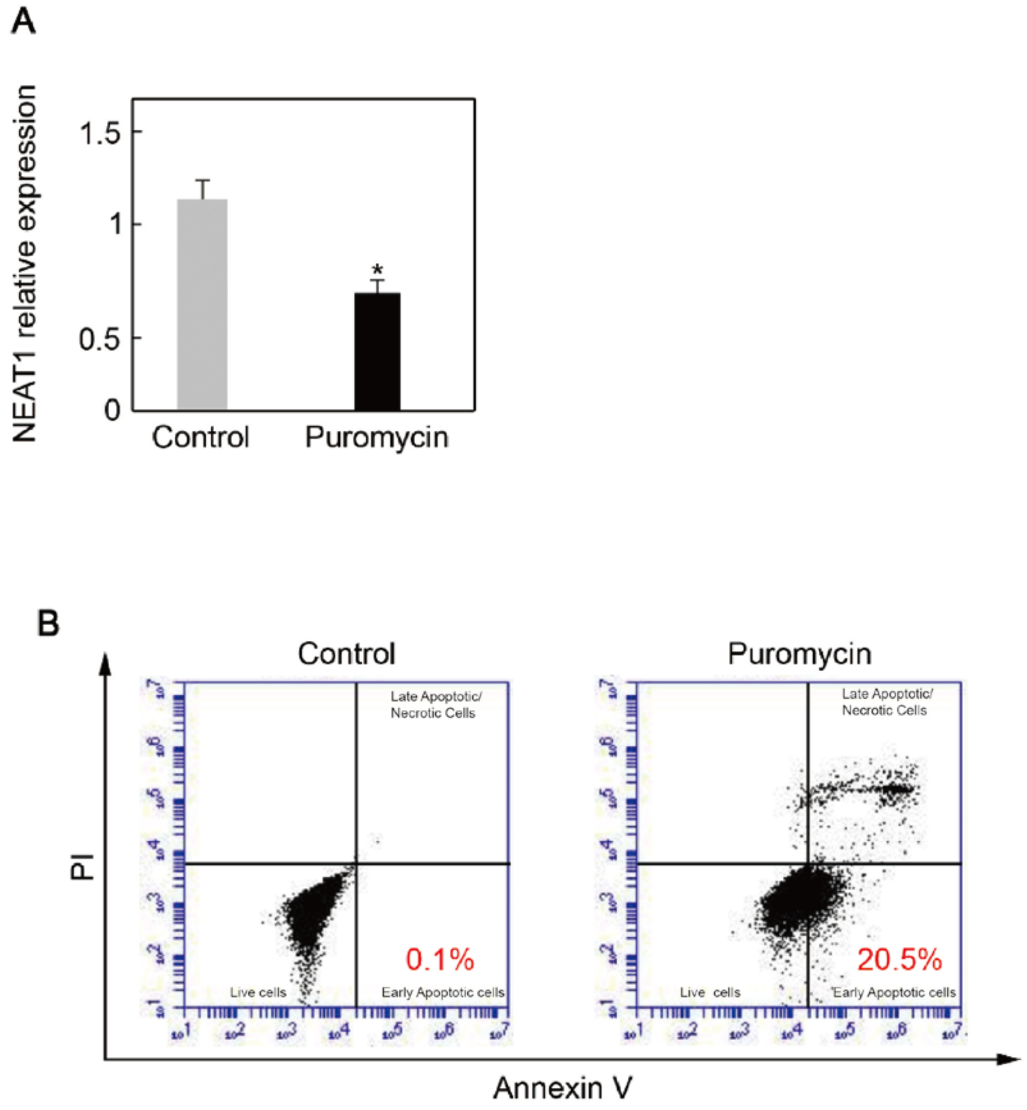

Supplementary Figure S1. Validation of correlations among puromycin-NEAT1-colorectal cancer. **A**. NEAT1 was down-regulated by puromycin treatment. HCT116 cells were treated with 7.5μM puromycin for 6 hours. NEAT1 expression was measured by qRT-PCR. **B**. Puromycin treatment induced HCT116 cells apoptosis. HCT116 cells were treated with 7.5μM puromycin for 6 hours. The percentage of apoptotic cells were determined by flow cytometry.

Cell culture

HCT116 human colorectal cancer cells were obtained from the Cell Bank of Chinese Academy of Sciences (Shanghai, China), and cultured in Dulbecco's

modified Eagle medium (Invitrogen, USA), supplemented with 10% fetal bovine serum (FBS; Invitrogen, USA), 50 U/mL penicillin, and 50 μg/mL streptomycin (Invitrogen, USA). Cells were maintained at 37°C in a humidified incubator at 5% CO2.

Puromycin treatment

HCT116 cells were seeded in 6cm dish 24 hours prior puromycin treatment for adherence. Cells were treated with 7.5μM puromycin (Sigma-Aldrich, USA) or PBS as control. After 6 hours treatment, cells were collected and subjected to the following experiments.

RNA isolation and qRT-PCR

HCT116 cells were collected after 6 hours puromycin treatment. Total RNAs were extracted using TRIzol reagent (Invitrogen, USA) according to the protocol of the manufacturer and reverse transcribed into cDNA using High Capacity cDNA Reverse Transcription Kit (Applied Biosystems, USA). Real-time PCR were performed using Power SYBR® Green PCR Master Mix (Applied Biosystems, USA). The following primer sets were used for Real-time PCR, and GAPDH were severed for normalization.
NEAT1-F: 5’- CTTCCTCCCTTTAACTTATCCATTCAC -3’,
NEAT1-R: 5’- CTCTTCCTCCACCATTACCAACAATAC -3’,
GAPDH-F: 5’- AGCCTCCCGCTTCGCTCTCT -3’,
GAPDH-R: 5’- GCGCCCAATACGACCAAATCCGT -3’.

Apoptosis analysis

HCT116 cells were collected after 6 hours puromycin treatment. Apoptotic cells were determined using eBioscience™ Annexin V-FITC Apoptosis Detection Kit (Invitrogen, USA) following indicated procedures. In brief, all cells were washed with PBS and re-suspended in binding buffer. Cells were stained with Annexin V-FITC for 10 minutes at room temperature and counterstained with propidium iodide. All cells were subjected to flow cytometry for apoptotic cell measurement.

Implementation of KEGG pathway enrichment analysis.

By repurposing microarray data of Cmap, we obtained 237 lncRNA signatures affected by 1262 drugs. All drugs and their affected lncRNAs were restored in LNCmap database. Based on these correlations among drugs and lncRNAs, users can study candidate medicine for a particular disease with correlated lncRNA data. In order to elucidate the use of our database, we provided two enrichment analysis algorithms: LSEA and ORA, to establish connections among diseases and drugs in terms of lncRNAs. Top ranked drugs in results may be used for directing the use of drug for disease. We took primary colorectal cancer for example, and verified 7 drugs (top 4 for LSEA results and top 3 for ORA results) were associated with colorectal

cancer or other cancer by searching literatures. Besides, we also implemented KEGG (http://www.genome.jp/kegg/) pathway analysis for these drug-induced lncRNAs according to reviewer's advice, procedure of pathway enrichment analysis was described as follows:

Firstly, we selected 7 drugs from LSEA and ORA results, top 4 drugs in LSEA results were apigenin, puromycin, cicloheximide and lycorine, top 3 drugs in ORA results were disulfiram, fendiline and sirolimus. For each drug, drug-induced lncRNAs were downloaded from LNCmap database, and we implemented pathway enrichment analysis for each affected lncRNA of this drug, enrichment analysis was calculated based on co-expressed protein-coding genes of this lncRNA by using SubpathwayMiner tools (http://www.bio-bigdata.net/SubpathwayMiner/). We calculated correlations between all lncRNAs and mRNAs that at least in 29 (80% of 36 primary colorectal cancer vs. normal samples) samples by pearson correlation coefficient, and set threshold of correlation to ±0.4, and adjusted p value to 0.01. For each lncRNA, all mRNAs met these two conditions were identified as co-expressed protein-coding genes and were merged into gene set for pathway enrichment analysis. The pathway enrichment analysis results were listed in Supplementary dataset (SI dataset 5). In the results, many significantly enriched pathways were dysregulated in colorectal cancer cells. For example, PI3K-Akt signaling pathway (path:04151) mutations were associated with longer time to local progression after radioembolization of colorectal liver metastases (Oncotarget. 2017 Feb 11). The MAPK signaling pathway (path:04010) regulated intrinsic resistance to the bromodomain and extra-terminal domain family proteins inhibitors in colorectal cancer (Clin Cancer Res. 2016 Sep 27). Besides, Luo et al reported that lncRNAs can influence downstream gene expression by inhibiting RNA polymerase (path:03020) and regulate gene expression as competing endogenous RNAs, and tissue and cancer-type specific lncRNAs could be useful prognostic markers (Oncotarget. 2017 Jan 18). Ye et al found that down regulated lncRNA CLMAT3 promotes the proliferation of colorectal cancer cells by targeting regulators of the cell cycle pathway (path:04110) (Oncotarget. 2016 Sep 13;7(37):58931-58938). Low proteasome (path:03050) activity was related to treatment resistance in colorectal cancer (Clin Cancer Res. 2016 Nov 1;22(21):5277-5286).
